# Accelerated and Severe Lupus Nephritis Benefits From M1, an Active Metabolite of Ginsenoside, by Regulating NLRP3 Inflammasome and T Cell Functions in Mice

**DOI:** 10.3389/fimmu.2019.01951

**Published:** 2019-08-14

**Authors:** Tsai-Jung Lin, Chung-Yao Wu, Pei-Yi Tsai, Wan-Han Hsu, Kuo-Feng Hua, Ching-Liang Chu, Yu-Chieh Lee, Ann Chen, Sheau-Long Lee, Yi-Jin Lin, Chih-Yu Hsieh, Shin-Ruen Yang, Feng-Cheng Liu, Shuk-Man Ka

**Affiliations:** ^1^Department of Pathology, National Defense Medical Center, Tri-Service General Hospital, Taipei, Taiwan; ^2^Graduate Institute of Life Sciences, National Defense Medical Center, Taipei, Taiwan; ^3^Department of Biotechnology and Animal Science, National Ilan University, Ilan, Taiwan; ^4^Graduate Institute of Immunology, National Taiwan University College of Medicine, Taipei, Taiwan; ^5^Department of Chemistry, R.O.C. Military Academy, Kaohsiung, Taiwan; ^6^Department of Internal Medicine, En Chu Kong Hospital, New Taipei City, Taiwan; ^7^Renal Care Joint Foundation, New Taipei City, Taiwan; ^8^Division of Rheumatology/Immunology and Allergy, Department of Internal Medicine, National Defense Medical Center, Tri-Service General Hospital, Taipei, Taiwan; ^9^Graduate Institute of Aerospace and Undersea Medicine, Department of Medicine, National Defense Medical Center, Taipei, Taiwan

**Keywords:** lupus nephritis, active metabolite of ginsenoside, NLRP3 inflammasome, regulatory T cell, autophagy

## Abstract

Chinese herbal medicines used in combination have long-term been shown to be mild remedies with “integrated effects.” However, our study provides the first demonstration that M1, an active metabolite of ginsenoside, exerted its dramatic therapeutic effects on accelerated and severe lupus nephritis (ASLN) mice, featuring acute renal function impairment, heavy proteinuria, high serum levels of anti-dsDNA, and high-grade, diffuse proliferative renal lesions. In the present study, NZB/WF1 mice were given injections of lipopolysaccharide to induce the ASLN model. M1 (30 mg/kg) was then administered to the mice by gavage daily, and the mice were sacrificed on week 3 and week 5 after the induction of disease. To identify the potential mechanism of action for the pure compound, levels of NLRP3 inflammasome activation in bone marrow-derived dendritic cells (BMDCs), podocytes and macrophages, and antigen-specific T cell activation in BMDCs were determined in addition to mechanistic experiments *in vivo*. Treatment with M1 dramatically improved renal function, albuminuria and renal lesions and reduced serum levels of anti-dsDNA in the ASLN mice. These beneficial effects with M1 treatment involved the following cellular and molecular mechanistic events: [1] inhibition of NLRP3 inflammasome associated with autophagy induction, [2] modulation of T help cell activation, and [3] induction of regulatory T cell differentiation. M1 improved the ASLN mice by blunting NLRP3 inflammasome activation and differentially regulating T cell functions, and the results support M1 as a new therapeutic candidate for LN patients with a status of abrupt transformation of lower-grade (mesangial) to higher-grade (diffuse proliferative) nephritis.

## Introduction

Lupus nephritis (LN) has six classes according to the level of severity of the renal pathology (1), and there is frequent transformation between the classes (2, 3). Acute induction of cellular and/or humoral autoimmune responses are considered a potential mechanism involved in pathological findings of a focal mild mesangial to diffuse proliferative LN (4–7), and the latter can be simulated by a mouse model of accelerated and severe LN (ASLN) in NZB/WF1 mice (8–12). Of note, episodes of bacterial or viral infections, as an environmental insult in genetically predisposed individuals may be associated with progression of systemic lupus erythematosus (SLE) to LN (13). High doses of corticosteroids and/or cytotoxic agents are main treatment of choice to control the severe renal condition (13, 14). Clinically, however, systemic side effects of these drugs, used either alone or in combination, are still a major concern (15, 16).

The NOD-like receptor family-pyrin domain containing 3 (NLRP3) inflammasome, which controls the activation of caspase-1 and in turn cleaves pro-IL-1β and pro-IL-18 to form mature IL-1β and IL-18 (17–19) has emerged as a key player in inflammatory responses and the induction of adaptive immunity, and it has garnered support as being important in promoting LN progression (19, 20). In addition, cumulative studies show that reactive oxygen species (ROS) act as second messengers whose signaling triggers NLRP3 inflammasome formation and activation (21, 22). Recently, we found that the uncontrolled production of IL-1β, IL-18, and ROS, and T cell activation are implicated in the activation of the NLRP3 inflammasome in a mouse model of accelerated and severe LN (ASLN) (8, 9, 11). Of note, NLRP3 inflammasome activation in podocytes of LN and other cells by lupus immune complex was identified, suggestive of the inflammasome contributing to the development of lupus, including LN (23–27). Therefore, it is conceivable that to inhibit NLRP3 inflammasome activation in a time-course manner may be beneficial to the subjects with LN or ASLN. Autophagy plays a vital role in maintaining cellular homeostasis (28, 29). Recently, autophagy has been shown to influence IL-1β secretion in macrophages (30, 31) and control the production of IL-1β by degrading pro-IL-1β (30, 32). Moreover, autophagy exerts an inhibitory effect on the NLRP3 inflammasome and can negatively regulate the innate immune response and inflammation (23, 33), for which various studies suggest that autophagy serves as a negative regulator of the NLRP3 inflammasome in the restoration of tissue homeostasis after damage in autoimmune diseases, including LN (34–36). Furthermore, several studies revealed that autophagy can protect against damage to podocytes (37–39).

Circulating dendritic cells (DCs) can localize within the glomeruli under IL-18 stimulation, thus triggering T cell activation and resultant renal damage in active LN (40). In addition, in patients with active and proliferative LN, T help (Th) and regulatory T (Treg) cells have been shown to play differential roles in the development and progression of the autoimmune condition (4, 41–43). Reduction of the number of Th1, Th2, or Th17 cells and the secretion of their cytokines as well as increase of the number of Treg cells and the secretion of their immunosuppressive cytokine can alleviate the severity of renal lesions in NZB/WF1 mice by inhibiting T cell-mediated transcription (42).

M1, a major absorbable intestinal bacterial metabolite of ginsenosides, is the major bioactive components of ginseng (44–46). It has been shown to have anti-inflammatory activity in adjuvant-induced rat arthritis (45) and zymosan-induced murine macrophage cells by reducing proinflammatory cytokines (47). M1 can negatively regulate NF-κB-mediated signaling pathway and inhibit pro-inflammatory cytokine production in tumor growth and chronic colitis (48–50). However, it remains unknown whether M1 can exert therapeutic effects on autoimmune nephritis, especially during its acute exacerbation.

In the present study, we showed that the pure compound exerted its beneficial effects in a murine ASLN model by inhibiting NLRP3 inflammasome, differentially regulating Th cells activation and Treg cells differentiation, and activating the sirtuin 3 (SIRT3)/autophagy axis. In agreement with these findings, an isobaric tag for relative and absolute quantitation (iTRAQ)-based proteomics analysis revealed down-regulated renal NLRP3 inflammasome activation-associated signaling pathways in M1-treated ASLN mice. The results suggest that M1 be a therapeutic candidate for LN patients with potential to develop an accelerated and deteriorated status, mimicking abrupt transformation of mild nephritis to higher-grade (severe) LN.

## Methods

### M1 Preparation

For the generation of the pure compound, M1, the leaves of the Chinese herb Panax notoginseng were prepared as described in our US patent (US7932057B2).

### Animal Model and Experiment Protocol

All animal experiments were performed with the ethical approval of the Institutional Animal Care and Use Committee of The National Defense Medical Center, Taiwan, in compliance with the NIH *Guide for the Care and Use of Laboratory Animals*. The ASLN mouse model was established in 8-week-old female NZB/WF1 mice (prior to autoantibody production) (purchased from Jackson Laboratory, ME, USA), with twice weekly intraperitoneal injections of lipopolysaccharide (LPS) (0.8 mg/kg body weight, Sigma, MO, USA) as described previously (8, 10). Seven days after the first injection of LPS, the mice were divided into 2 groups of 8 mice each and were given either M1 (50 mg/kg body weight) or vehicle (corn oil) daily via oral gavage. Age-matched female NZB/WF1 mice injected with normal saline served as normal controls. The mice were sacrificed at week 3 and week 5, respectively, after the induction of disease.

### Clinical and Histopathological Evaluation

Albuminuria was evaluated by the ratio of urine albumin to urine creatinine (Cr) (urine albumin/Cr), as described previously (8–11). Serum levels of blood urea nitrogen (BUN) and Cr were determined as described previously (9, 10). Formalin-fixed and paraffin-embedded renal sections were stained with hematoxylin and eosin (H&E), for which the scoring of renal histopathological alterations was determined and glomerulonephritis activity was scoring for LN patients as described previously (51). For immunohistochemistry (IHC), methyl Carnoy's solution fixed and paraffin-embedded tissue sections were stained with against F4/80^+^ (Serotec, NC, USA) or CD3^+^ (pan T cell; Dako, Glostrup, Denmark), and frozen sections were stained with against CD11c^+^ (BioLegend, CA, USA), as described previously (11). For immunofluorescence (IF) staining of LC3B, cultured cells were stained with against LC3B (Sigma). For scoring of the number of CD3^+^-, F4/80^+^-, or CD11c^+^-positive cells, a quantitative image analysis software (Pax-it; Paxcam, IL, USA) was used as previously described (8).

### Antigen-Specific T Cell Activation With Bone Marrow-Derived Dendritic Cells (BMDCs)

The mononucleocytes from mouse BM were isolated from tibias and femurs of 8-week-old female NZB/WF1 mice. The cells were cultured in presence of granulocyte-macrophage colony stimulating factor (GM-CSF; 10 ng/ml) (Invitrogen, CA USA) for 6 days to induce differentiation of mononucleocytes into DCs, as described previously (52). For antigen-specific T cell activation, BMDCs were with incubated with LPS (100 ng/ml) for 24 h. The cells then pulsed with OVA_323−339_ peptide (1 μg/ml, Genomics, Taiwan)-stimulated CD4^+^ T cells, that were derived from lymph node cells of OT-II transgenic mice (kindly provided by Dr. C. Lowell, University of California, USA). Then antigen-specific CD4^+^ T cell activation were measured uptake of [3H]-thymidine (Amersham Pharmacia Biotech, New Jersey, USA) as described below. The ratios of the total cell numbers for BMDCs vs. CD4^+^ T cells in 1:2 for T cells proliferation assay and IL-17A production, and in 1:8 for IFN-γ production were adopted, respectively.

### Culture of Podocytes and Macrophages

Conditionally immortalized mouse podocytes were obtained from Dr. S. J. Shankland (Department of Medicine, University of Washington, USA) and were cultured at 37°C in the presence of IFN-γ (Roche, NJ, USA), and differentiated for 13 to 15 days at 37°C in the absence of IFN-γ, as described previously (53). The murine macrophage cell line J774A.1 was purchased from the American Type Culture Collection (USA) and was maintained as described previously (54).

### Western Blot Analysis

Protein lysates were run on SDS-PAGE gels, as described previously (8, 53, 55). Anti-NLRP3 (AdiopGen, CA, USA), IL-1β, caspase-1, VEGF, TGF-β, β-actin (Santa Cruz, Tx, USA), p-IκB, SIRT3 (Cell Signaling, MA, USA), LC3B (Sigma), Atg5 (MBL, Japan), COX-2, or NADPH p47^phox^ (Santa Cruz) antibodies were used.

### [^3^H] Thymidine Incorporation

Proliferation of CD3^+^ T cells in mouse splenocytes, and antigen-specific CD4^+^ T cell activation with BMDCs were measured uptake of [3H]-thymidine (Amersham Pharmacia Biotech) using a TopCount (Packard/PerkinElmer, Boston, MA) as described previously (52). Briefly, mouse splenocytes were isolated and cultured in 96-well flat-bottomed plates coated overnight with anti-mouse CD3 antibody (BD Biosciences). After 48 h, the cultures were pulsed with 1 μCi of [^3^H] thymidine (Amersham International, Buckinghamshire, UK) and harvested 16 hr later, and was measured using a TopCount (PerkinElmer Life Sciences, Palo Alto, CA, USA).

### Flow Cytometry

Mouse splenocytes were double-stained with FITC-conjugated anti-mouse CD3^+^ (17A2; pan T cells) or CD4^+^ (GK1.5; helper T cells) and phycoerythrin-conjugated anti-mouse CD69^+^ (H1.2F3) antibodies (all from BD Biosciences, CA, USA). For intracellular staining, briefly, splenocytes were cultured in the presence of 20 ng/ml phorbol nyristate acetate, 1 mM ionomycin, and 4 mM monensin (all from Sigma) for 6 h. The cells were stained with FITC-conjugated anti-mouse CD4 (BD Biosciences) and allophycocyanin-conjugated anti-mouse IFN-g (XMG1.2) or IL-4 (11B11) antibodies (BD Biosciences), as described previously (9, 53). Cells were stained for Treg cells using the Mouse Regulatory T cell Staining Kit (eBioscience) according to the manufacturer's instructions. Maturation of BMDCs was determined by the upregulation levels of CD11c^+^ and CD80^+^ as described previously (48). The serum levels of IL-1β, IFN-γ, IL-12p70, TNF-α, IL-6, MCP-1 and IL-10 were determined using a cytometric bead array mouse inflammation kit (BD Biosciences) according to the manufacturer's protocol. A flow cytometer (FACSCalibur, BD Biosciences) was used for these experiments, as described previously (9, 54).

### ELISA and Enzyme Activity Assay

The levels of IL-1β, IL-17A, and INF-γ in supernatant of cultured cells were measured using commercial ELISA kits (R&D Systems, MN, USA), while caspase-1 activity was measured using a caspase-1 fluorometric assay kit (R&D systems) according to the manufacturer's instructions. Serum levels of anti-dsDNA antibodies were detected using an anti-mouse dsDNA ELISA kit (Alpha Diagnostic, USA) according to the manufacturer's instructions.

### Determination of ROS Levels

Three different techniques were employed for renal tissues, sera, BMDCs and podocytes. *In situ* superoxide anion production was determined in renal tissues by dihydroethidium (DHE; Sigma) labeling and quantified, as described previously (9, 53). ROS levels in serum and renal tissues were measured by a lucigenin-enhanced chemiluminescence assay, as described previously (8, 9). Intracellular ROS production in BMDCs and podocytes was measured by detecting the fluorescence intensity of 2′, 7′-dichlorofluorescein diacetate (Invitrogen), as described previously (52). N-acetyl-L-cysteine (NAC) is a ROS scavenger, and used it as a positive control for evaluating the inhibitory effect of M1 on ROS production in BMDCs and podocytes.

### Real-Time PCR Assay

RNA was extracted by REzol (Protech Technology, Taipei, Taiwan), and SYBR Green RT-PCR Reagents Kit (Applied Biosystems, MA, USA) was used as described previously (9, 55). The PCR primer pairs used for analysis were as follows: mouse IL-1β forward: 5′- CCAGGATGAGGACATGAGCACC-3′ and reverse: 5′- TTCTCTGCAGACTCAAACTCCAC-3′; mouse IL-18 forward: 5′-ACTGTACAACCGGAGTAATACGG-3′ and 5′-TCCATCTTGTTGTGTCCTGG-3′; mouse GAPDH forward: 5′-TCCGCCCCTTCTGCCGATG-3′ and reverse: 5′-CACGGAAGGCCATGCCAGTGA-3′; mouse T-bet forward: 5′-TCCCATTCCTGTCCTTCA-3′ and reverse: 5′-GCTGCCTTCTGCCTTTC-3′; mouse GATA-3 forward: 5′-ACCACGGGAGCCAGGTATG-3′ and reverse: 5′-CGGAGGGTAAACGGACAGAG-3′.

### Statistical Analysis

Data are presented as means ± SEM, and were analyzed using one-way ANOVA and subsequent Scheffe's test. A *p-*value < 0.05 was considered statistically significant for each of the experiments.

## Results

### M1 Improved Renal Conditions and Reduced Serum Levels of Anti-dsDNA Autoantibodies

**Figure 1:**
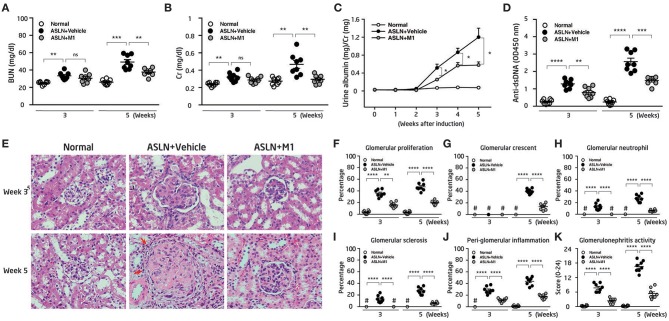
Renal function, albuminuria, renal pathology, and anti-dsDNA antibodies. **(A)** Levels of serum BUN and **(B)** serum Cr. **(C)** Time-course studies of albuminuria levels. **(D)** Levels of serum anti-dsDNA autoantibody. **(E)** H&E staining. Arrows indicate mononuclear leukocyte infiltration. Original magnification x400. **(F–J)** Scoring of H&E. **(K)** Scoring of glomerulonephritis activity. The data are the means ± SEM for 8 mice per group. ASLN, accelerated and severe lupus nephritis. BUN, blood urea nitrogen. Cr, creatinine. ^*^p < 0.05, ^**^p < 0.01, ^***^p < 0.005, and ^****^p < 0.001. #Not detectable. ns, no significant difference.

Vehicle-treated ASLN (ASLN+Vehicle) mice developed abnormal renal function as demonstrated by elevated serum levels of BUN and Cr at both week 3 and week 5 after the induction of disease, compared with normal control mice (Figures 1A,B). In contrast, this effect was significantly inhibited in M1-treated ASLN (ASLN+M1) mice at week 5, although at week 3, there was no detectable difference in the levels of renal function between ASLN+Vehicle and ASLN+M1 mice (Figures 1A,B). As shown in Figure 1C, ASLN+Vehicle mice showed albuminuria as demonstrated by elevated ratios of urine albumin/Cr at week 3 and week 5, compared with normal control mice. However, the ASLN+M1 mice presented significantly decreased levels of albuminuria. Similarly, significantly lowered serum levels of anti-dsDNA autoantibodies were seen in ASLN+M1 mice, compared with those of ASLN+Vehicle mice, at both week 3 and week 5 (Figure 1D). By light microscopy, early at week 3, the ASLN+Vehicle mice started to develop significant glomerular proliferation and interstitial inflammation (Figures 1E–J). At week 5, the ASLN+Vehicle mice revealed characteristic severe lesions, including intrinsic cell proliferation, cellular crescent formation, neutrophil infiltration, and sclerosis in the glomerulus as well as interstitial inflammation that featured peri-glomerular mononuclear leukocyte infiltration (Figures 1E–J). In contrast, ASLN+M1 mice showed greatly improved renal lesions at week 3, and at week 5, only focal mesangial proliferation and mild interstitial inflammation was identified in the mice (Figures 1E–J). As shown in Figure 1K, although significantly high glomerulonephritis activity scores were observed in the ASLN+Vehicle mice compared with normal control mice, this effect was inhibited in ASLN+M1 mice at both the week 3 and week 5. By IHC, ASLN+Vehicle mice showed profound infiltration of CD3^+^ T cells (Supplementary Figures 1A,B), F4/80^+^ monocyte/macrophages (Supplementary Figures 1C,D) and CD11c^+^ DCs (Supplementary Figures 1E,F) in the glomeruli and renal interstitial tissues at both week 3 and week 5. However, these effects of mononuclear leukocyte infiltration in the kidney were significantly inhibited in ASLN+M1 mice (Supplementary Figures 1A–F).

### M1 Reduced Serum Cytokine Levels

**Figure 2:**
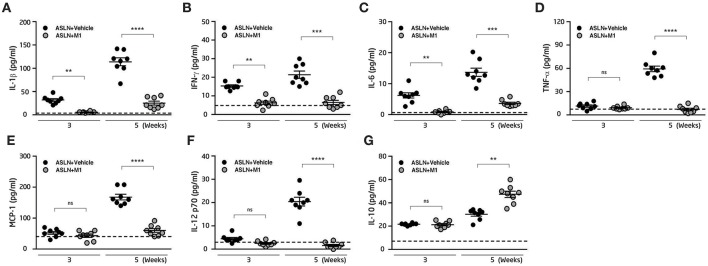
Serum inflammatory cytokine expression. Serum levels of **(A)** IL-1β, **(B)** IFN-γ, **(C)** IL-6, **(D)** TNF-α, **(E)** MCP-1 **(F)** IL-12 p70, and **(G)** IL-10. The horizontal dashed line indicates the mean of levels from normal control mice. The data are the means ± SEM for 8 mice per group. ASLN, accelerated and severe lupus nephritis. ^**^p < 0.01, ^***^p < 0.005, and ^****^p < 0.001. ns, no significant difference.

M1 has been shown to have anti-inflammatory activity and in arthritis rat models and murine macrophages (45, 50), we then measured serum levels of cytokines in the mice. As shown in Figures 2A–C, significantly increased serum levels of IL-1β, IFN-γ, and IL-6 were observed in ASLN+Vehicle mice, compared with those of the normal control mice at both week 3 and week 5, but these effects were greatly inhibited in ASLN+M1 mice. In addition, serum levels of TNF-α, MCP-1, and IL-12p70 were increased in the ASLN+Vehicle mice at week 5, but significantly reduced serum levels of these cytokines were seen in the ASLN+M1 mice (Figures 2D–F). Moreover, ASLN+M1 mice produced significantly higher IL-10 levels than ASLN+Vehicle mice at week 5, although there was no difference in serum levels of this cytokine early at week 3 (Figure 2G).

### M1 Differentially Regulated Th Cell Activation and Treg Cell Differentiation

**Figure 3:**
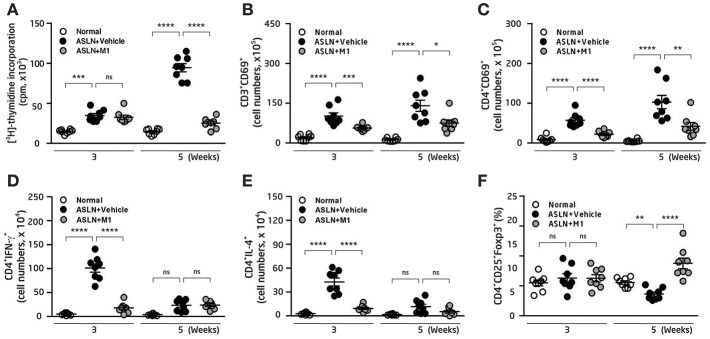
T-cell function. **(A)** T-cell proliferation in splenocytes by thymidine uptake analysis. **(B)** CD3^+^CD69^+^ T cells; **(C)** CD4^+^CD69^+^ T cells; **(D)** CD4^+^IFNγ^+^ T cells; **(E)** CD4^+^IL-4^+^ T cells; **(F)** CD4^+^CD25^+^Foxp3^+^ Treg cells, in splenocytes, by flow cytometry. The data are the means ± SEM for 8 mice per group. ASLN, accelerated and severe lupus nephritis. ^*^p < 0.05, ^**^p < 0.01, ^***^p < 0.005, and ^****^p < 0.001. ns, no significant difference.

Dysfunction of Th subsets is highly pertinent to the development of LN (4, 42, 43). Furthermore, Th and Treg cells have been shown to involve active LN in patients (4, 43). We then examined the effects of M1 on Th cell activation and Treg cell differentiation. As demonstrated by thymidine uptake assay in splenocytes, significantly increased T cell proliferation was observed in the ASLN+Vehicle mice compared with normal control mice at both week 3 and week 5 (Figure 3A). However, M1 treatment greatly reduced T cell proliferation near the levels that were seen in normal control mice at week 5 (Figure 3A). Moreover, the total cell numbers of CD3^+^CD69^+^ cells and CD4^+^CD69^+^ cells in the splenocytes of the ASLN+M1 mice were significantly decreased compared with those of the ASLN+Vehicle mice at both week 3 and week 5 (Figures 3B,C). For further identification of the Th bias, the analysis with intracellular staining showed that the total cell numbers of Th cells (CD4^+^ cells) that expressed IFN-γ or IL-4 were greatly increased in the splenocytes of ASLN+Vehicle, and this effect was inhibited in the ASLN+M1 mice at week 3 (Figures 3D,E). In addition, significantly increased total cell numbers of CD4^+^CD25^+^Foxp3^+^ Treg cells were seen in the ASLN+M1 mice at week 5, compared with those of ASLN+Vehicle mice, although there was no difference in the total cell numbers of Treg cells among the three groups of mice at week 3 (Figure 3F).

### M1 Inhibited ROS Production and NLRP3 Inflammasome Activation

#### ROS Production in BMDCs, Podocytes, and Renal Tissues

**Figure 4:**
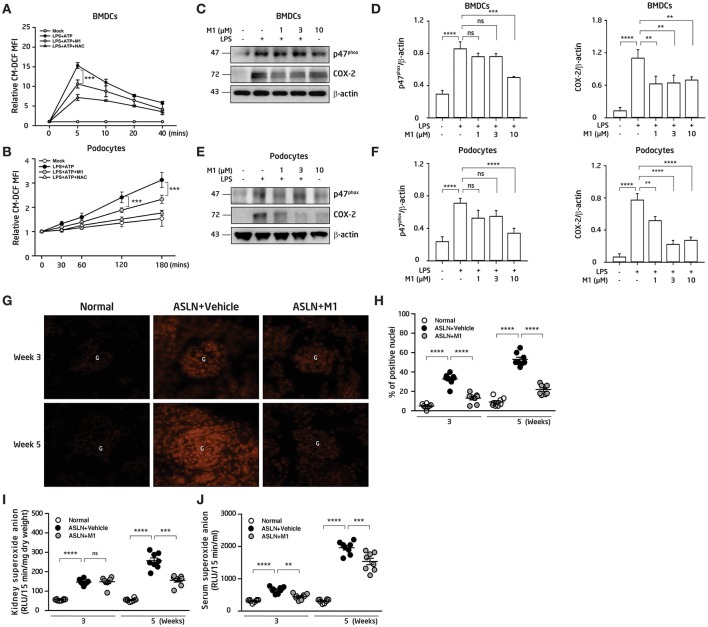
ROS production. **(A)** ROS levels in BMDCs. The cells were incubated with M1 (10 μM) or NAC (10 mM; a ROS scavenger) for 30 min, then treated with or without 100 ng/ml LPS for 5.5 h and ATP (5 mM). **(B)** ROS levels in podocytes. The cells were incubated with M1 (10 μM) or NAC (10 mM; a ROS scavenger) for 30 min, then treated with or without 1 μg/ml LPS for 5.5 hr and ATP (5 mM). The ROS production were measured by 2′, 7′ -dichlorofluorescein diacetate after incubated with ATP. **(C)** NADPH oxidase p47^phox^ (p47^phox^) and COX-2 protein levels in BMDCs by Western blotting and **(D)** semiquantitative analysis. BMDCs were incubated with M1 (10 μM) for 30 min, then treated with or without 100 ng/ml LPS for 6 h. **(E)** NADPH oxidase p47^phox^ (p47^phox^) and COX-2 protein levels in podocytes by Western blotting and **(F)** semiquantitative analysis. The podocytes were incubated with M1 (10 μM) for 30 min, then treated with or without 1 μg/ml LPS for 6 h. The data are expressed as the means ± SEM for three separate experiments, and each experiment was performed in triplicate. **(G)** *In situ* renal ROS production with DHE staining and **(H)** scoring. Original magnification, 400×. ROS levels in **(I)** renal tissues and **(J)** serum. The data are the means ± SEM for 8 mice per group. ASLN, accelerated and severe lupus nephritis. BMDC, bone marrow-derived dendritic cells. NAC, N-acetyl-L-cysteine. G, glomerulus. DHE, dihydroethidium. ^**^p < 0.01, ^***^p < 0.005, and ^****^p < 0.001. ns, no significant difference.

Overproduction of ROS contributes to the activation of NLRP3 inflammasome (21, 22). DCs present antigens and prime T-cell responses in various inflammatory conditions, and have a critical role to play for the evolution of LN (4, 43). First, the effect of M1 on the production of ROS in BMDCs and podocytes was evaluated. As shown in Figures 4A,B, M1 reduced the ATP-mediated production of ROS in LPS-primed and ATP-activated BMDCs and podocytes, respectively, and the finding suggests that the compound inhibited the activation of NLRP3 inflammasome in these cells. Moreover, M1 significantly decreased the protein levels of p47^phox^ and COX-2 at a dose-dependent manner in LPS-primed BMDCs (Figures 4C,D) and podocytes (Figures 4E,F), respectively.

Next, compared with normal control mice, although the levels of DHE fluorescence detected by an *in situ* analysis were greatly elevated in the glomeruli and some renal tubules in the ASLN+Vehicle mice, at week 3 to week 5, this effect was significantly inhibited in the ASLN+M1 mice (Figures 4G,H). In parallel, ROS levels in the kidney of the ASLN+Vehicle mice were significantly increased compared with those of normal control mice at week 3 and continued to rise up to week 5, but M1 significantly reduced the ROS levels at week 5 in the ASLN+M1mice (Figure 4I). Furthermore, M1 significantly decreased the ROS levels in the sera of ASLN+M1 mice compared with those of ASLN+Vehicle mice at both week 3 and week 5, although these levels were higher than normal control mice at week 5 (Figure 4J).

#### NLRP3 Inflammasome Activation in BMDCs, Podocytes, Macrophages and Renal Tissues

**Figure 5:**
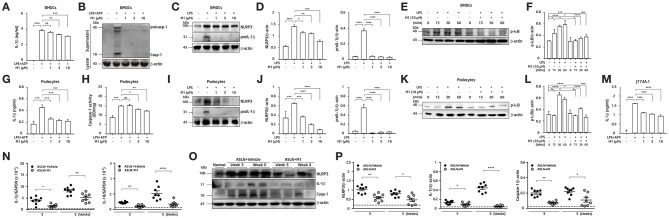
NLRP3 inflammasome activation. **(A–F)** BMDCs. **(G–L)** Podocytes. **(M)** Mouse J774A.1 macrophages. The BMDCs were incubated with the indicated concentrations of M1 for 30 min, and then incubated with 100 ng/ml LPS for 5.5 h and 5 mM ATP for 30 min. **(A)** IL-1β secretion in BMDCs was measured by ELISA, and **(B)** pro-caspase-1 (procasp-1) and secretion of caspase-1 (Casp-1) in supernatant of BMDCs were measured by Western blotting. **(C,D)** NLRP3 and proIL-1β protein levels and **(E,F)** p-IκB protein levels in BMDCs by Western blotting after incubated with M1 for 30 min, and then incubated with 100 ng/ml LPS for 6 h or the indicated times. **(G)** IL-1β secretion and **(H)** caspase-1 activity in podocytes were measured by ELISA after incubated with the indicated concentrations of M1 for 30 min, and then incubated with 100 ng/ml LPS for 5.5 h and 5 mM ATP for 30 min. **(I,J)** NLRP3 and proIL-1β and **(K,L)** p-IκB protein levels in podocytes by Western blotting after incubated with M1 for 30 min, and then incubated with 1 μg/ml LPS for 6 h or the indicated times. **(M)** IL-1β secretion in J774A.1 macrophages was measured by ELISA. The macrophages were incubated with the indicated concentrations of M1 for 30 min, and then incubated with 1 μg/ml LPS for 5.5 h and 5 mM ATP for 30 min. The data are expressed as the means ± SEM for three separate experiments, and each experiment was performed in triplicate. **(N)** Renal IL-1β and IL-18 mRNA levels by qPCR analysis. **(O)** Renal expression of NLRP3, IL-1β and caspase-1 (Casp-1) by Western blotting and **(P)** semiquantitative analysis. The horizontal dashed line indicates the mean of levels from normal control mice. The data are the means ± SEM for 8 mice per group. The horizontal dashed line indicates the mean of levels from normal control mice. BMDCs, bone marrow-derived dendritic cells. ASLN, accelerated and severe lupus nephritis. ^*^p < 0.05, ^**^p < 0.01, ^***^p < 0.005, and ^****^p < 0.001. #Not detectable. ns, no significant difference.

As shown in Figure 5, treated with M1, the LPS-primed BMDCs were found to have decreased ATP-mediated IL-1β secretion (Figure 5A) and caspase-1 activation (Figure 5B) in a dose-dependent manner.Additionally, M1 significantly reduced the expression levels of NLRP3 and proIL-1β in LPS-primed BMDCs (Figures 5C,D). Furthermore, M1 significantly inhibited the expression levels of p-IκB in LPS-primed BMDCs in a time-dependent manner (Figures 5E,F).

Activation of NLRP3 inflammasomes in podocytes (20) or macrophages (8, 17, 56) also contributes to the disease progression of LN. ATP-mediated IL-1β secretion (Figure 5G) and caspase-1 activation (Figure 5H) were both likely reduced by M1 in LPS-primed mouse podocytes in a dose-dependent manner. Again, M1 suppressed the expression of NLRP3 and proIL-1β in LPS-primed podocytes (Figures 5I,J). As shown in Figures 5K,L, treatment with M1 also significantly inhibited the protein levels of p-IκB in LPS-primed podocytes in a time-dependent manner. The inhibitory effect of M1 on the NLRP3 inflammasome was also tested using a mouse macrophage cell line J774A.1, as M1 could inhibit ATP-mediated IL-1β secretion in LPS-primed macrophages (Figure 5M). Together, these findings suggest that the compound inhibited both the priming and activating signals of the NLRP3 inflammasome in these cells.

Although mRNA levels of IL-1β and IL-18 (Figure 5N), and protein expression levels of NLRP3, IL-1β and caspase-1 (Figures 5O,P) were significantly increased in renal tissues of ASLN+Vehicle mice compared with normal control mice, these effects were inhibited in ASLN+M1 mice at both week 3 and week 5.

### M1 Inhibited NLRP3 Inflammasome via Autophagy Induction in BMDCs and Podocytes

**Figure 6:**
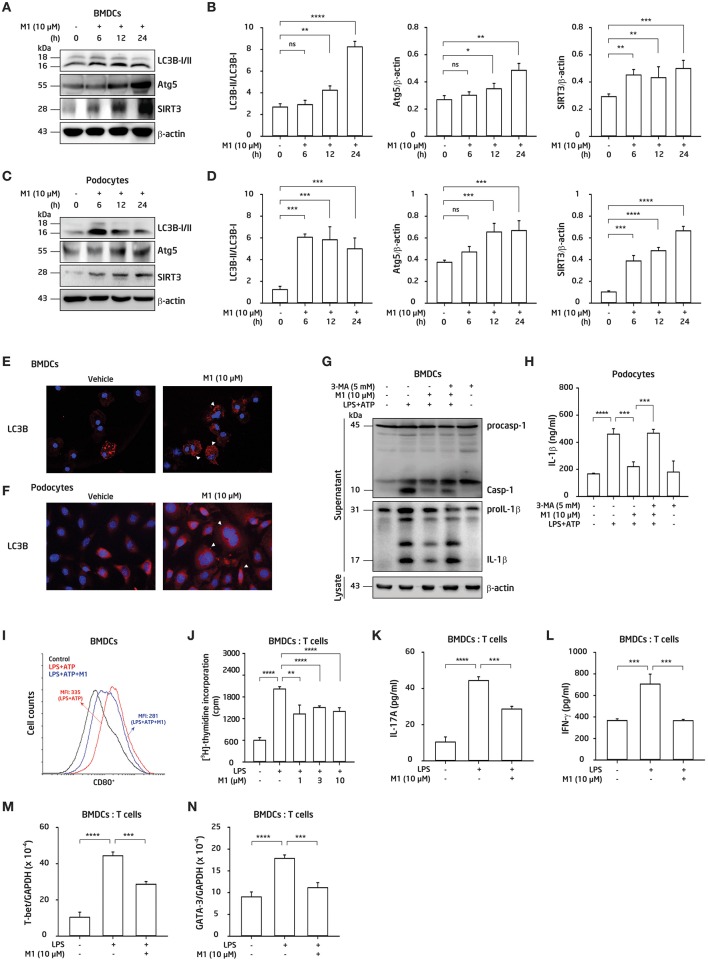
M1 inhibited the NLRP3 inflammasome via autophagy induction, and regulated adaptive immunity. **(A,B,E,G,I–K)** BMDCs. **(C,D,F,H)** Podocytes. Protein expression levels of LC3B, Atg5 and SIRT3 by Western blotting after incubated for 0–24 h with the indicated concentrations of M1 in **(A,B)** BMDCs and **(C,D)** podocytes. LC3B expression by immunofluorescence staining in **(E)** BMDCs and **(F)** podocytes. Both the cells incubated with M1 (10 μM) or vehicle (DMSO; 0.2%) for 6 h. Arrowhead indicates the positive staining. **(G)** Protein expression levels of caspase-1 (Casp-1) and IL-1β in supernatant of BMDCs by Western blotting, and **(H)** IL-1β secretion in podocytes by ELISA, after incubated for 5 mM 3-MA (autophagy inhibitor) for 30 min, then with 10 μM M1 for 30 min followed by 100 ng/ml (BMDCs) or 1 μg/ml (podocytes) LPS for 5.5 h, and 5 mM ATP for 30 min. **(I)** Expression levels of CD80^+^ (within gated CD11c^+^ cells) determined by flow cytometry in BMDCs from 8-week-old female NZB/WF1 mice, which were incubated with M1 (10 μM) 30 min, then 100 ng/ml LPS for 24 h and 5 mM ATP for 30 min. **(J)** OT-II antigen-specific T cell proliferation was measured by ^3^H-thymidine incorporation. The ratio of the total cell numbers for BMDCs vs. CD4^+^ T cells in 1:2 was used. The BMDCs were incubated with M1 (1, 3 or 10 μM) 30 min and LPS (100 ng/ml) for 24 h, the collected supernatants were cocultured with OT-II CD4^+^ T cells pulsed with OVA_323−339_ peptide (1 μg/ml) for further 24 h incubation, then added [^3^H] thymidine for detecting the proliferation by β-counters. **(K)** IL-17A production, and **(L)** IFN-γ production were assayed by ELISA. The ratios of the total cell numbers for BMDCs vs. CD4^+^ T cells in 1:2 for IL-17A and in 1:8 for IFN-γ were adopted, respectively. The BMDCs were incubated with M1 (10 μM) 30 min and LPS (100 ng/ml) for 24 h, the collected supernatants were cocultured with OT-II CD4^+^ T cells pulsed with OVA_323−339_ peptide (1 μg/ml) for further 3 days incubation. Supernatants were collected for those proteins analysis. **(M,N)** mRNA levels of T-bet and GATA-3 were measured by qPCR analysis. The BMDCs were incubated with M1 (10 μM) 30 min and LPS (100 ng/ml) for 24 h, the collected supernatants were cocultured with OT-II CD4^+^ T cells pulsed with OVA_323−339_ peptide (1 μg/ml) for further 3 days incubation. Cells were collected for RNA extraction and qPCR analysis. The data are expressed as the means ± SEM for three separate experiments, and each experiment was performed in triplicate. BMDC, bone marrow-derived dendritic cells; 3-MA, 3-methyladenine, an autophagy inhibitor. MFI, mean fluorescence intensity. ^*^p < 0.05, ^**^p < 0.01, ^***^p < 0.005, and ^****^p < 0.001. ns, no significant difference.

We next investigated whether autophagy was involved in the protective effect of M1 in BMDCs and podocytes. BMDCs or podocytes were incubated with M1 in a time-course manner. As shown in Figure 6, M1 enhanced autophagy in BMDCs (Figures 6A,B) and podocytes (Figures 6C,D), as evidenced by the increase in the LC3B-II/LC3B-I ratios and Atg5 levels, indicating an enhancement of the autophagic response in these cells by M1 treatment. Besides, SIRT3 is also involved in the process of autophagy induction (31, 57), and can inhibit the production of IL-1β by reducing pro-IL-1β and ROS production (31, 32). As shown in Figure 6, M1 treatment increased the expression levels of SIRT3 in both BMDCs (Figures 6A,B) and podocytes (Figures 6C,D). Furthermore, M1 treatment significantly increased the expression of LC3B as shown in increased puncta formation in M1-treated BMDCs (Figure 6E) and podocytes (Figure 6F), respectively. To test whether the inhibitory effect of M1 on the NLRP3 inflammasome was attributable to autophagy induction. We found that 3-MA, an autophagy inhibitor, reversed the inhibitory effect of M1 on caspase-1 activation and IL-1β secretion (Figure 6G) in LPS-primed and ATP-activated BMDCs. Additionally, 3-MA also reversed the inhibitory effect of M1 on IL-1β secretion in LPS-primed and ATP-activated podocytes (Figure 6H). These results suggest that M1 suppressed the activation of the NLRP3 inflammasome in BMDCs and podocytes by enhancing the induction of autophagy.

### M1 Modulated BMDCs-Mediated Antigen-Specific T Cell Proliferation and Activation

In view that M1 inhibited the NLRP3 inflammasome activation in BMDCs (Figure 5) and regulated T cell function *in vivo* (Figure 3), next, we examined the effect of M1 on BMDCs activation and the resultant T cell responses. As shown in Figure 6I, significantly decreased percentages of CD11c^+^CD80^+^ DCs were seen in M1-treated LPS-primed BMDCs. Moreover, an OVA-antigen specific T cell proliferation assay showed that LPS-primed BMDCs showed an increased proliferation of CD4^+^ T cells, and this effect was greatly inhibited by treatment with M1 (Figure 6J). Furthermore, M1 decreased the production of IL-17A (Figure 6K) and IFN-γ (Figure 6L) by activated T cells. M1 significantly inhibited the mRNA levels of T-bet (Figure 6M) and GATA3 (Figure 6N) in the activated T cells, which were co-incubated with M1-treated LPS-primed BMDCs. Collectively, these results suggest that M1 abrogated the ability of the cells to induce antigen-specific T cell proliferation and activation.

### Down-Regulated Renal NLRP3 Inflammasome Activation-Associated Signaling Pathways

Totally quantified 3,816 proteins were identified by the iTRAQ-based proteomics analysis in renal tissues of normal control, ASLN+Vehicle and ASLN+M1 mice. In order to systematically understanding the effects of M1 on the ASLN mice, gene set enrichment analysis was used to evaluate the biologically enriched pathways/networks in the comparisons of ASLN+M1 mice vs. ASLN+Vehicle mice. The data showed that systemic lupus erythematosus pathway was significantly down-regulated and suggest the several signaling pathways associated with NLRP3 inflammasome activation on MAPK, ECM-receptor interaction, cell adhesion molecules, Toll-like receptor, FcγR-mediated phagocytosis and leukocyte transendothelial migration may all contribute to the mechanism of action of the potential renoprotective effects of M1 in the treated ASLN mice (Supplementary Table S1).

## Discussion

**Figure 7:**
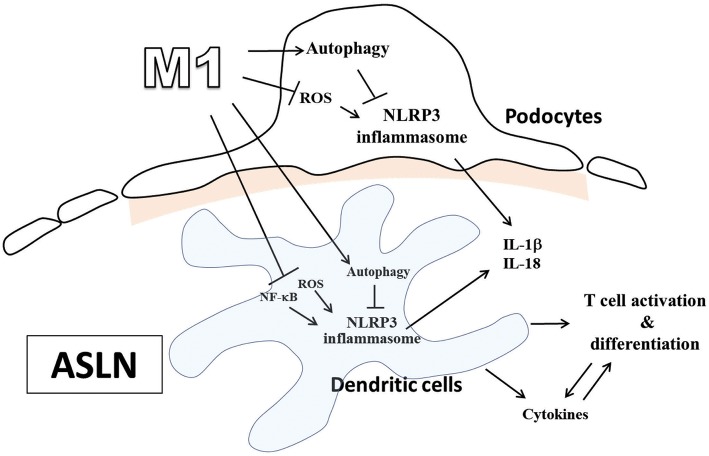
Schematic representation for the plausible mechanism of action for the therapeutic effects of M1 on ASLN. ASLN, accelerated and severe lupus nephritis.

It is well-known that Chinese herbal medicines used in combination have long-term been shown to be mild remedies with “integrated effects.” However, we first demonstrated that M1 exerted its dramatic therapeutic effects in ASLN mice, characterized by acute renal function impairment, heavy proteinuria, high serum levels of anti-dsDNA, and high-grade, diffuse proliferative renal lesions. We showed these beneficial effects of M1 clearly correlated with the following: [1] inhibition of NLRP3 inflammasome associated with autophagy induction in renal tissues, BMDCs or podocytes, [2] modulation of Th cell activation, and [3] induction of Treg cell differentiation in ASLN (Figure 7).

The NLRP3 inflammasome has broad implications for a variety of kidney diseases due to its connection with immunity, proinflammatory cytokines and autophagy (58). We previously showed that inhibiting the activation of the NLRP3 inflammasome by reducing ROS production and NF-κB activation significantly ameliorated renal injury in the mouse ASLN model (8). In the present study, we found that M1 inhibited protein expression of p-IκB in BMDCs and podocytes, which were LPS-primed, suggesting that one of the major effects M1 exerted could be the inhibition of pro-IL-1β production through its suppression of NF-κB activation. We also demonstrated that M1 was able to reduce protein levels of p47^phox^ and COX-2 at a dose-dependent manner in both the LPS-primed cells. In addition, we showed that decreased ROS production was seen in sera and the kidney in ASLN+M1 mice and in BMDCs and podocytes that were treated with M1. Consistent with our findings, Chen et al. demonstrated that M1 inhibits IL-1β maturation and reduces NLRP3 inflammasome activation in adipose tissue treated with high glucose (56), and many reports have revealed that the major functional components of ginsenoside have antioxidant properties (47, 50). Together, we infer that reduced ROS generation by M1 might explain the resultant decreased NLRP3 inflammasome activation.

Furthermore, we found that significantly increased autophagic responses in mouse BMDCs that were treated with M1. Autophagy has been shown to influence IL-1β secretion in macrophages (30, 31) and control the production of IL-1β by degrading pro-IL-1β (30, 32) to inhibit NLRP3 inflammasome activation, and this process can also negatively regulate innate immune response and inflammation (23, 33). In the present study, we demonstrated that 3-MA, an autophagy inhibitor, reversed the inhibitory effect of M1 on NLRP3 inflammasome activation in mouse BMDCs, suggesting that M1 suppressed the activation of the NLRP3 inflammasome through autophagy induction, as a potential mechanism of action for the pure compound in ASLN. Although both M1+LPS- and LPS-treated cells showed increased LC3B protein levels, there was no significant difference in the expression levels of the protein (data not shown). The possible explanation for this effect could be that LPS itself can induce autophagy activation in various cells, including immune cells and somatic cells (59, 60).

In the present study, we demonstrated that treatment with M1 significantly inhibited the activation of the NLRP3 inflammasome in the BMDCs of ASLN+M1 mice. We also demonstrated that M1 administration significantly inhibited the infiltration of the mononuclear leukocytes, including DCs, macrophages and T cells into the kidney of ASLN+M1 mice. Here, we found that M1 administration significantly decreased the maturation of BMDCs, and inhibited the proliferation and differentiation of their induced antigen-specific CD4^+^ T cells by the BMDCs. These findings suggest that M1 regulated the adaptive immune response mediated by T cell functions through DCs. Of note, although some measurements were statistically significant, biological implications of such differences may need to be further investigated, including why minimal effect of M1 on IL-1β secretion in BMDCs (Figure 5A) and lowered percentage of activated T cells (CD69^+^ T cells) of splenocytes in the ASLN+M1 mice (Figures 3B,C) were observed. In addition, IL-10 is an important mediator of Treg cell suppression, and IL-10 production by Treg cells plays a key anti-inflammatory role (61). Induction of IL-10-secreting Treg cells by anti-CD3 antibody treatment in a lupus-prone model in mice can inhibit the activation of effector T cells (Th1, Th2 and Th17), whereby improving the renal condition in mice (62). In the present study, we demonstrated that treatment with M1 significantly inhibited T cell proliferation and activation and increased the proportion of Treg cells and production of IL-10 in ASLN mice. These combined effects of M1, which simultaneously inhibited T cell activation but promoted Treg cell activity, may be crucial in terms of the mechanism of action for M1 to exert its therapeutic effect on this murine ASLN model. Besides, the kinetics of Th1 (IFNγ^+^) or Th2 (IL-4^+^) and Treg (CD25^+^Foxp3^+^) is different, which might make it difficult to explain the *in vivo* phenotype, and it is worth further investigation to address this issue.

The activation of NLRP3 inflammasome has been implicated in the pathogenesis of podocyte injuries and the development of proteinuria in patients with LN (20). In the present study, we showed that treatment with M1 significantly inhibited the activation of NLRP3 inflammasome in podocytes, through autophagy induction. Taken together, these results suggest that M1 protected podocyte injury in mice with ASLN by enhancing autophagy, thus protecting against NLRP3 inflammasome activation.

In conclusion, treatment with M1 dramatically improved the ASLN mice by inhibiting NLRP3 inflammasome activation in combination with autophagy induction and differentially regulating T cell functions, and the results support M1 as a new therapeutic candidate for LN patients with a status of abrupt transformation of lower-grade (mild mesangial) to higher-grade (diffuse proliferative) nephritis.

## Data Availability

All datasets generated for this study are included in the manuscript and the Supplementary Files.

## Author Contributions

All authors were involved in drafting the article or revising it critically for important intellectual content and all authors approved the final version to be published. S-MK and F-CL had full access to all of the data in the study and take responsibility for the integrity of the data and the accuracy of the data analysis.

### Conflict of Interest Statement

The authors declare that the research was conducted in the absence of any commercial or financial relationships that could be construed as a potential conflict of interest.

## Supplementary Material

Supplementary Figure 1 Renal infiltration of CD3^+^ T cells, F4/80^+^ macrophages and CD11c^+^ dendritic cells. **(A,B)** CD3^+^ T cells, **(C,D)** F4/80^+^ macrophages and **(E,F)** CD11c^+^ dendritic cells by immunohistochemistry and quantitative analysis. The arrow indicates CD3^+^ T-cell, F4/80^+^ macrophages or CD11c^+^ dendritic cell infiltration. Original magnification, 400 ×. The data are the means ± SEM for 8 mice per group. ASLN, accelerated and severe lupus nephritis. ^*^*p* < 0.05, ^**^*p* < 0.01, and ^***^*p* < 0.005. #Not detectable.
