# Supplementary material for: Accelerated and Severe Lupus Nephritis Benefits From M1, an Active Metabolite of Ginsenoside, by Regulating NLRP3 Inflammasome and T Cell Functions in Mice
Source: Front Immunol. 2019 Aug 14;10:1951. doi: 10.3389/fimmu.2019.01951 (PMC6702666; doi:10.3389/fimmu.2019.01951)
Supplement: Supplementary file 1 [file Table_1.DOCX]

**Supplementary Table S1**. **Forty-four enriched KEGG gene sets (23 pathways down-regulated and 21 pathways up-regulated by M1) from ASLN mice**.

| **Category I** | **Category II** | **Pathway name** | **Normalized enrichment score** | | |
| --- | --- | --- | --- | --- | --- |
|  |  |  | ASLN+Vehicle/  Normal | ASLN+M1/  Normal | ASLN+M1/ ASLN+Vehicle |
| Metabolism | Global and overview maps | Fatty acid metabolism | -2.091^*†^ | 0.739 | 2.814^*†^ |
|  | Carbohydrate metabolism | Glycolysis / Gluconeogenesis | -1.475^*†^ | 0.883 | 1.667^*†^ |
|  |  | Citrate cycle (TCA cycle) | -2.520^*†^ | -1.140 | 2.864^*†^ |
|  |  | Pyruvate metabolism | -2.026^*†^ | -1.090 | 2.295^*†^ |
|  |  | Glyoxylate and dicarboxylate metabolism | -1.082 | 1.201 | 2.287^*†^ |
|  |  | Propanoate metabolism | -2.706^*†^ | -1.000 | 3.247^*†^ |
|  |  | Butanoate metabolism | -2.498^*†^ | 0.873 | 3.256^*†^ |
|  | Energy metabolism | Nitrogen metabolism | -1.697^*†^ | -1.053 | 2.256^*†^ |
|  | Lipid metabolism | Glycerolipid metabolism | 0.900 | 1.148 | 1.521^*†^ |
|  | Amino acid metabolism | Alanine, aspartate and glutamate metabolism | -1.789^*†^ | -0.643 | 2.437^*†^ |
|  |  | Glycine, serine and threonine metabolism | -2.168^*†^ | 0.638 | 2.725^*†^ |
|  |  | Valine, leucine and isoleucine degradation | -2.989^*†^ | -1.514^*^ | 3.620^*†^ |
|  |  | Lysine degradation | -1.673^*†^ | 0.755 | 1.887^*†^ |
|  |  | Arginine and proline metabolism | -1.449^*†^ | 0.705 | 1.860^*†^ |
|  |  | Tyrosine metabolism | -1.122 | 0.595 | 1.570^*†^ |
|  |  | Tryptophan metabolism | -2.306^*†^ | 0.760 | 3.326^*†^ |
|  | Metabolism of other amino acids | beta-Alanine metabolism | -1.592^*†^ | 1.132 | 2.632^*†^ |
|  |  | Glutathione metabolism | -1.633^*†^ | -1.004 | 1.993^*†^ |
|  | Glycan biosynthesis and metabolism | Other glycan degradation | 1.466^*†^ | -1.240 | -1.786^*†^ |
|  | Metabolism of cofactors and vitamins | Pantothenate and CoA biosynthesis | -1.274^†^ | -0.982 | 1.567^*†^ |
|  |  | One carbon pool by folate | -0.889 | 1.160 | 1.813^*†^ |
|  | Xenobiotics biodegradation and metabolism | Metabolism of xenobiotics by cytochrome P450 | -1.438^†^ | -1.060 | 1.517^*†^ |
|  |  | Drug metabolism - cytochrome P450 | -1.641^*†^ | -0.994 | 1.939^*†^ |
| Genetic information processing | Translation | Ribosome | -1.899^*†^ | -3.677*† | -2.833^*†^ |
| Environmental information processing | Signal transduction | MAPK signaling pathway | 1.501^*†^ | 1.377 | -1.451^*†^ |
|  | Signaling molecules and interaction | ECM-receptor interaction | 2.072^*†^ | 1.723^*†^ | -2.046^*†^ |
|  |  | Cell adhesion molecules (CAMs) | 1.770^*†^ | 1.736^*†^ | -1.981^*†^ |
| Cellular processes | Transport and catabolism | Lysosome | 1.741^*†^ | 1.392^*^ | -1.740^*†^ |
|  |  | Peroxisome | -1.768^*†^ | 0.779 | 2.376^*†^ |
|  | Cellular community - eukaryotes | Focal adhesion | 1.721^*†^ | 1.257 | -1.832^*†^ |
|  | Cell motility | Regulation of actin cytoskeleton | 1.715^*†^ | 1.434^*^ | -1.649^*†^ |
| Organismal systems | Immune system | Hematopoietic cell lineage | 1.814^*†^ | 1.758^*†^ | -2.047^*†^ |
|  |  | Complement and coagulation cascades | 2.218^*†^ | 2.137^*†^ | -1.765^*†^ |
|  |  | Toll-like receptor signaling pathway | 1.490^*†^ | 1.430 | -1.443^*†^ |
|  |  | Cytosolic DNA-sensing pathway | 1.453^†^ | 1.182 | -1.612^*†^ |
|  |  | Fc gamma R-mediated phagocytosis | 1.729^*†^ | 1.576^*†^ | -1.426^*†^ |
|  |  | Leukocyte transendothelial migration | 1.677^*†^ | 1.738^*†^ | -1.561^*†^ |
| Human diseases | Cancers: Overview | Pathways in cancer | 1.396^*†^ | 1.177 | -1.468^*†^ |
|  | Cancers: Specific types | Small cell lung cancer | 1.475^*†^ | 0.969 | -1.650^*†^ |
|  | Immune diseases | Systemic lupus erythematosus | 1.455 | 1.014 | -1.660^*†^ |
|  | Cardiovascular diseases | Hypertrophic cardiomyopathy (HCM) | 1.936^*†^ | 1.428 | -1.827^*†^ |
|  |  | Arrhythmogenic right ventricular cardiomyopathy (ARVC) | 1.636^*†^ | 1.280 | -1.693^*†^ |
|  |  | Dilated cardiomyopathy | 1.801^*†^ | 1.376 | -1.871^*†^ |
|  | Infectious diseases: Parasitic | Leishmaniasis | 1.618^*†^ | 1.735^*†^ | -1.575^*†^ |

^*^ Nominal *p*-value <0.05; ^†^ False discovery rate *q*-value <0.25.
