# Supplementary figures and images for: Accelerated and Severe Lupus Nephritis Benefits From M1, an Active Metabolite of Ginsenoside, by Regulating NLRP3 Inflammasome and T Cell Functions in Mice
Source: Front Immunol. 2019 Aug 14;10:1951. doi: 10.3389/fimmu.2019.01951 (PMC6702666; doi:10.3389/fimmu.2019.01951)

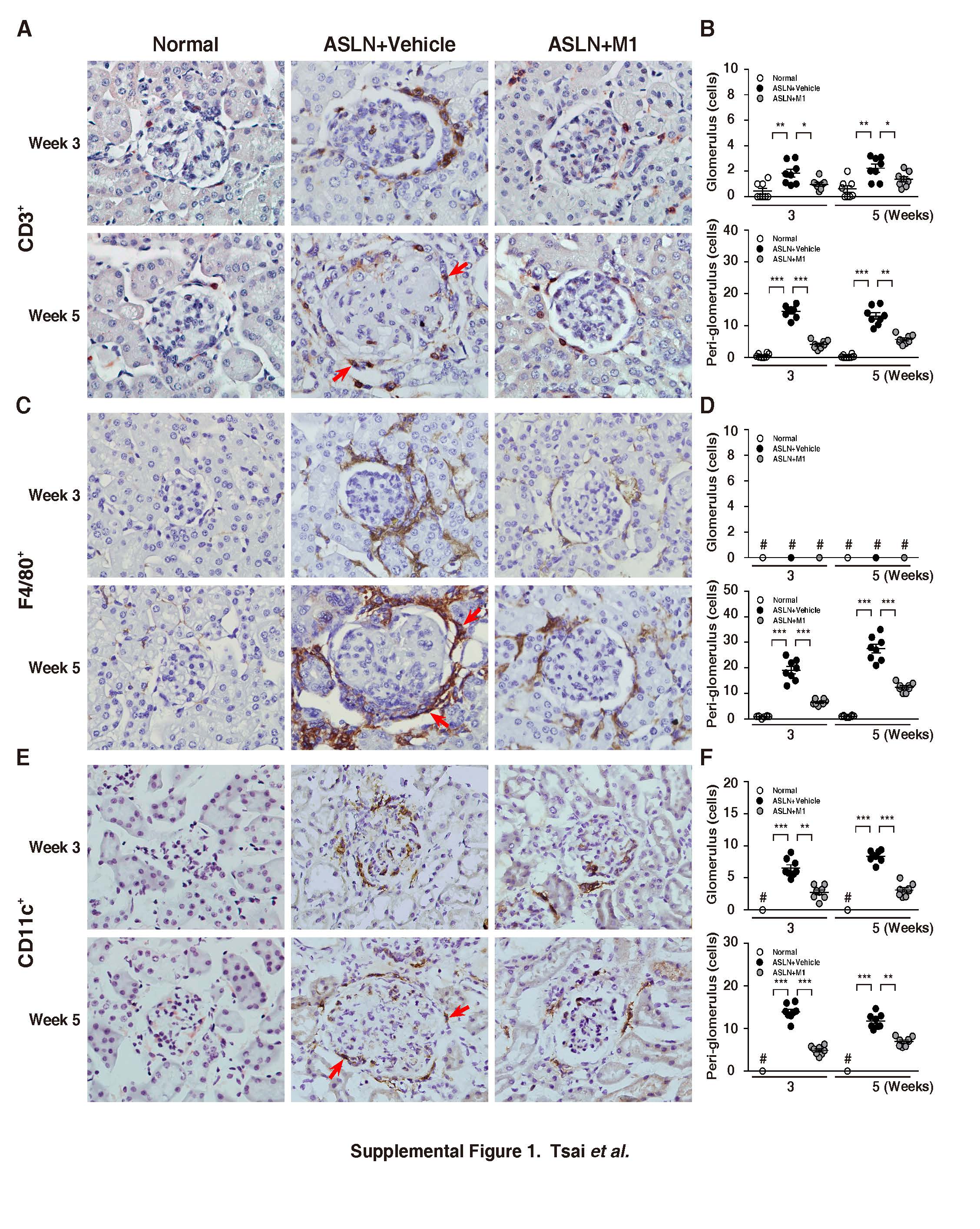

Supplement: Supplementary Figure 1 — Renal infiltration of CD3+ T cells, F4/80+ macrophages and CD11c+ dendritic cells. (A,B) CD3+ T cells, (C,D) F4/80+ macrophages and (E,F) CD11c+ dendritic cells by immunohistochemistry and quantitative analysis. The arrow indicates CD3+ T-cell, F4/80+ macrophages or CD11c+ dendritic cell infiltration. Original magnification, 400 ×. The data are the means ± SEM for 8 mice per group. ASLN, accelerated and severe lupus nephritis. *p < 0.05, **p < 0.01, and ***p < 0.005. #Not detectable. [file Image_1.JPEG]
